# Exploring the association between loneliness, work environment, and depressive symptoms: evidence from young Korean workers in the Seoul Metropolitan Area

**DOI:** 10.3389/fpubh.2025.1593957

**Published:** 2025-05-30

**Authors:** Geon Lee, Chulwoo Kim

**Affiliations:** ^1^Department of Public Administration, Hanyang University, Seoul, Republic of Korea; ^2^Department of Public Administration, Gachon University, Seongnam, Republic of Korea

**Keywords:** workplace mental health, job satisfaction, workplace bullying, depressive symptoms, loneliness, young workers

## Abstract

**Background:**

Mental health in the workplace is an emerging public health concern, particularly for young workers who may experience challenges that contribute to psychological distress. This study examined the associations between loneliness, work environment, and depressive symptoms among young workers in Seoul, South Korea.

**Methods:**

A cross-sectional survey was conducted among young workers in Seoul to assess workplace conditions, job satisfaction, workplace bullying, and depressive symptoms. Negative binomial regression analysis was used to evaluate the associations between selected workplace factors, including job-major alignment, commute time, workplace bullying, job satisfaction, and mental health outcomes.

**Results:**

A negative association was found between job satisfaction and depressive symptoms, and a positive association was found between workplace bullying and depressive symptoms. Job–major alignment and commute time were not significantly related to depressive symptoms. The findings also highlighted a meaningful relationship between workplace social connections and loneliness among young workers.

**Conclusion:**

These results suggest that workplace conditions, such as job satisfaction and social climate, may be important factors in understanding depressive symptoms among younger working populations. Given that the data were limited to young workers in Seoul, future research should adopt a longitudinal design and conduct comparative studies across different regions and countries to gain a more comprehensive understanding of workplace mental health.

## 1 Introduction

Mental health in the workplace has emerged as a critical public health concern, as organizations increasingly acknowledge its relevance to employee well-being and productivity (1–4). Workplace stressors, such as long hours, job insecurity, and high-performance demands, have been associated with higher levels of depressive symptoms, anxiety, and burnout. These mental health challenges are, in turn, linked to reduced work performance, greater absenteeism, and higher turnover rates (5, 6). Young workers appear particularly susceptible to workplace-related mental health issues, as they frequently encounter conditions such as employment instability, uncertainty regarding career prospects, and elevated performance expectations—factors that have been correlated with psychological distress (7–9). In highly competitive work environments, extended working hours, limited job security, and low levels of task autonomy may further contribute to worsening mental well-being (10–13). These concerns are particularly pronounced in South Korea, where a high-pressure occupational culture and strong societal expectations are commonly linked with heightened stress levels among young employees (14–16).

Workplace stress is a significant social issue in South Korea, particularly among younger employees. Many young employees report high levels of stress, which appears to be associated with internal workplace dynamics and general differences in values, leading some to consider job changes. Workplace bullying is also a major concern, with approximately 49% of affected employees reporting thoughts of resignation due to mental health challenges (17). According to the Ministry of Employment and Labor, the number of officially reported workplace bullying cases has reached approximately 12,000 annually (18), indicating that workplace stress remains prevalent. This increasing psychological burden is reflected in a sharp increase in antidepressant prescriptions. Between 2014 and 2023, the number of antidepressant prescriptions in South Korea rose from 14.4 million to approximately 23.3 million, with the largest increase observed among individuals between 20 and 30 years old (19).

The correlation between workplace stress and overall health is further supported by findings from the 2024 Korean Social Survey, which reported that 62.1% of respondents experienced workplace-related stress. Moreover, occupational stress is not confined to corporate environments, as similar patterns have been observed in the public sector. For example, the number of elementary school employees seeking medical treatment for depressive symptoms nearly doubled from 4,819 in 2020 to 9,468 in 2023 (20). These trends indicate that occupational stress may extend beyond specific professional groups to become a broader societal concern.

In the absence of targeted responses to these challenges, there may be long-term implications not only for individual careers but also for the broader stability of the workforce. Therefore, companies are encouraged to prioritize mental health in addition to productivity. Gaining insights into how workplace conditions are associated with younger employees’ mental well-being can help inform workplace policies and public health strategies.

Understanding how workplace conditions relate to mental health requires a structured theoretical perspective. This study draws on the Job Demands–Resources (JD-R) model to examine workplace stress and the Cognitive Activation Theory of Stress (CATS) to explore how workplace bullying and loneliness may be linked to stress responses. Together, these frameworks help to interpret how occupational factors are linked to depressive symptoms among young workers.

The JD-R model outlines how an imbalance between job demands and available resources is associated with psychological strain (21, 22). Factors such as long commutes, excessive workload, and job dissatisfaction are considered job-related stressors that may contribute to mental exhaustion. In contrast, elements such as job autonomy, social support, and fair compensation are regarded as resources potentially associated with lower stress levels (23, 24). Employees who report limited work engagement, particularly when their academic training is not aligned with their job responsibilities, may be more likely to experience burnout and depressive symptoms (25).

The CATS describes how persistent exposure to stressors such as workplace bullying and chronic loneliness can be linked to adverse mental health outcomes (26, 27). Repeated experiences of verbal abuse, social exclusion, or prolonged isolation may be associated with enduring stress responses, reduced resilience, and lowered self-esteem, which in turn are correlated with higher levels of anxiety and depressive symptoms (28–30). When loneliness becomes pervasive beyond the workplace, it may reinforce negative cognitive patterns and reduce the ability to cope with stress (31, 32). In addition, a workplace culture that permits bullying may exacerbate emotional distress and intensify depressive symptoms among employees (33, 34).

Empirical evidence highlights the complex interplay between workplace conditions and mental health, particularly among younger populations. Loneliness has been consistently associated with elevated depressive symptoms, especially in young adults navigating social and professional transitions (35, 36). In the workplace context, job satisfaction has been shown to have a robust inverse relationship with depressive symptoms, suggesting that individuals who perceive their jobs more positively tend to report lower psychological distress (37, 38). Conversely, exposure to workplace bullying, such as verbal abuse, exclusion, and unjust treatment, has been linked to a higher risk of depressive symptoms and emotional exhaustion (39, 40). However, much of this literature focuses on Western contexts or broader adult populations, leaving a gap in the understanding of how these dynamics manifest among young urban workers in East Asian societies such as South Korea. This study aimed to address this gap by examining how loneliness, job satisfaction, and bullying are associated with depressive symptoms among young workers in Seoul.

By integrating these theories with empirical evidence, this study provides a comprehensive framework for understanding how workplace factors contribute to mental health challenges. Recognizing these dynamics is essential for developing workplace policies that prioritize employee well-being and prevent work-related depressive symptoms.

## 2 Materials and methods

### 2.1 Study population and survey design

We analyzed data collected by the Seoul Institute through the Seoul Young Adult Panel Study, first conducted in 2021, to examine the life behaviors of young adults residing in Seoul, the capital city of South Korea. This study covered various aspects, including social relationships, employment status, educational background, economic activity, life satisfaction, emotional well-being, and subjective health. The study population consisted of only Korean nationals aged 18–35 years and living in Seoul at the end of November 2021. The survey employed a probability sampling method, selecting households through multistage cluster-stratified sampling based on the sample area, followed by selecting eligible participants within each of these households. Various computer-assisted data collection modes were utilized, including computer-assisted web interviewing, tablet-assisted personal interviewing, and computer-assisted mobile interviewing. The survey was conducted between 20 August and 29 December 2022. Initially, a sample of 9,184 eligible individuals was selected; however, only 5,194 participated in the survey, resulting in a response rate of 56.6%. All participants received a cash incentive in Korean currency equivalent to approximately USD 13. For our analysis, we used a subsample of 3,219 employed respondents, excluding unemployed individuals. The study protocol involving human participants received approval from the research planning and coordination committee of the Seoul Institute. All ethical standards for research involving human subjects were strictly followed, including obtaining informed consent and ensuring participant anonymity. Participation in the survey was entirely voluntary. Respondents had the option to skip any questions they preferred not to answer and could decline participation or withdraw from the study at any point. All responses were collected anonymously and kept confidential.

### 2.2 Main variables

The primary outcome variable in this study was depressive symptoms, assessed using the Center for Epidemiologic Studies Depression Scale (CES-D) (41, 42). The original CES-D is a 20-item instrument designed to measure depressive symptoms in the general population, with each item rated on a four-point scale ranging from zero (“rarely or none of the time”) to three (“most or all of the time”). This study utilized the Korean-language version of the CES-D-11, which includes the following items: “poor appetite” (item two), “as good as others” (item four), “depressive symptoms” (item six), “too much effort” (item seven), “troubled sleep” (item 11), “loneliness” (item 14), “unfriendly people” (item 15), “enjoy life” (item 16), “sad” (item 18), “others dislike me” (item 19), and “cannot get going” (item 20) (43). Two items were reverse-coded before summation to yield a total score ranging from zero to 33. Previous research by Park and Kim (43) confirmed the CES-D-11’s reliability, unidimensionality, and measurement invariance between the general population and parents of individuals with cerebral palsy in Korea.

One of the primary independent variables in this study was loneliness, measured using the UCLA Loneliness Scale (Version 3) (44), which consists of 20 items rated on a four-point scale ranging from one to four. Nine items were reverse-coded before summing all items to produce a total score ranging from 20 to 80. Workplace-related factors were also examined. Commute time was assessed based on respondents’ self-reported travel time to work and categorized into a binary variable (“less than 30 min” vs. “30 min or more”). Job satisfaction was measured using a single-item question: “How satisfied are you with your job?” on a five-point Likert scale ranging from one (“not satisfied at all”) to five (“very satisfied”). Job-major alignment was evaluated using the question, “To what extent does your job match your major?” rated on a three-point scale from one (“not matched at all”) to three (“perfectly matched”). Experience of workplace bullying was assessed as a binary variable (yes versus no). Workplace size was determined based on a closed-ended question with four response categories: “1–4 employees,” “5–299 employees,” “300 or more employees,” and “Do not Know” (DK). The DK responses were treated as missing values in the analysis. All variables were derived from respondents’ self-reports. A common limitation of regression analyses using self-reported data is the potential for common method bias. To assess this concern, we conducted Harman’s one-factor analysis and found that the largest proportion of variance explained by a single variable was 19.63%, which is well below the 50% threshold. This suggests that common method bias was not a serious concern in our analysis.

### 2.3 Covariates

Several covariates that might influence the outcome were considered, including sex, age, marital status, household composition, education, homeownership, and subjective income. Age was initially collected as an open-ended response in the survey, but was subsequently categorized into four groups: 18–25, 26–29, 30–32, and 33–35. Marital status was classified into three categories: “not married,” “married,” and “divorced or separated.” Household composition was measured as a dichotomous variable, distinguishing between individuals living alone and those cohabiting. Education level was categorized into three groups: “high school or less,” “college,” and “graduate level.” Subjective income level was originally measured on a 10-point scale in the survey, but was recoded into five levels for analytical purposes.

### 2.4 Statistical analysis

While 3,219 cases were available for analysis, item nonresponse occurred because respondents did not answer all survey questions. The number of item nonresponse cases varied across the questions. There are two primary methods for handling item nonresponse behavior: imputation and listwise deletion. In this study, we employed the listwise deletion method to conservatively estimate regression coefficients, minimize type I errors, and reduce the likelihood of overstating statistical significance. The analysis is conducted in three stages. First, to examine whether depressive levels differed across groups, we compared CES-D-11 mean scores among groups using Student’s t-tests for binary groups (for example, sex) and analysis of variance (ANOVA) for categorical variables with more than two groups (for example, marital status). Second, we conducted a multivariate regression analysis to identify workplace-related factors and covariates influencing depressive symptoms using negative binomial regression models. A negative binomial regression model was selected because the outcome variable (CES-D-11 score) was count-based, did not follow a normal distribution, and exhibited overdispersion, as indicated by the substantial difference between the mean and variance. Although we also considered the Poisson regression model, this model assumes equality between the mean and variance of the outcome, which was not supported by our data. Examination of the mean and variance of the total CES-D-11 score revealed that the variance was considerably greater than the mean. Furthermore, we assessed model fit using the Pearson chi-square goodness-of-fit test, which confirmed that the negative binomial regression model provided the best fit for our data.

## 3 Results

### 3.1 Socio-demographic characteristics of participants

Table 1 presents the socio-demographic characteristics of the study participants. In terms of sex distribution, 44.17% of the participants were male, while 55.83% were female. Regarding age, the largest proportion of participants fell within the 30–32 age group (34.2%), followed by those aged 26–29 (29.2%), whereas the smallest proportion was among individuals aged 18–25 (16.43%). With respect to marital status, most participants (86.09%) were not married, while 13.31% were married, and only 0.6% were divorced or separated. Household composition indicated that 62.83% of the participants lived alone, whereas approximately 37% cohabited with others. Regarding educational attainment, most participants (67.54%) had completed college, followed by high school graduates (24.85%) and those with graduate-level education (7.61%). In terms of homeownership, 52.77% of the participants owned a home, whereas approximately 47% did not. The distribution of subjective income levels indicated that approximately 40% of participants perceived their income level as intermediate, nearly 32% rated their income as low or very low, and approximately 23% assessed their income as high or very high.

**Table 1:** Socio-demographic characteristics of the workers (*N* = 3,219).

Variables	*N*	Proportion (%)
Gender		
Male	1,382	44.17
Female	1,747	55.83
Age		
18–25	514	16.43
26–29	915	29.24
30–32	1,070	34.20
33–36	630	20.13
Marital status		
Not married	2,037	86.09
Married	315	13.31
Divorced or separated	14	0.59
Household		
Single	1,966	62.83
Cohabit	1,163	37.17
Education		
High school or less	588	24.85
College	1,598	67.54
Graduate level	180	7.61
Homeownership		
Owner	726	52.77
Non-owner	811	47.23
Subjective income level		
1–2 (very low)	227	9.68
3–4 (low)	1,000	24.64
5–6 (intermediate)	1,249	39.43
7–8 (high)	613	23.25
9–10 (very high)	40	3.00

### 3.2 Association between baseline variables and depressive symptoms

Table 2 presents the bivariate associations between the baseline variables and depressive symptoms as measured by CES-D-11 scores. A significant relationship was observed between loneliness and CES-D-11 scores. To examine this association, loneliness was categorized into four groups: 34 or less, 35–49, 50–64, and 65 or more. The results indicated a significant difference in CES-D-11 scores across these groups (*p* < 0.001). The highest mean CES-D-11 score (30.97) was recorded for individuals with the highest loneliness index (65 or higher), followed by the 50–64 group (24.04), the 35–49 group (18.37), and 34 or less (14.39). These findings indicate a positive correlation between loneliness and depressive symptoms. No significant association was found between commute time and CES-D-11 scores. However, job satisfaction exhibited a strong inverse relationship with depressive symptoms (*p* < 0.001), as higher levels of job satisfaction were correlated with lower CES-D-11 scores. Similarly, a significant association was found between job-major alignment and CES-D-11 scores (*p* < 0.001), with mean scores of 18.64 for those reporting no such alignment, 17.88 for those with some alignment, and 17.17 for those with perfect alignment.

**Table 2:** Unadjusted association between baseline variables and depressive symptoms.

Variables	CESD-11 Mean (SD)	*P*-value
Loneliness		<0.001
34 <	14.39 (3.58)	
35–49	18.37 (5.16)	
50–64	24.04 (6.80)	
65=>	30.97 (8.46)	
Commute time (min)		0.640
30 <	18.07 (6.17)	
30 =>	17.96 (6.38)	
Work satisfaction		<0.001
1 (not at all)	23.81 (7.38)	
2	20.66 (6.81)	
3	18.60 (6.14)	
4	16.78 (5.56)	
5 (very)	16.61 (6.71)	
Job–major alignment		<0.001
Not at all	18.64 (6.57)	
Somewhat	17.88 (6.03)	
Perfect	17.17 (5.92)	
Experience of workplace bullying		<0.001
Yes	20.83 (7.19)	
No	17.72 (7.19)	
Number of employees		<0.001
1–4	18.47 (6.66)	
5–299	18.19 (6.37)	
300 or more	17.16 (5.50)	
Gender		<0.001
Male	17.30 (6.07)	
Female	18.60 (6.33)	
Age		0.725
18–25	18.02 (6.20)	
26–29	18.15 (6.49)	
30–32	17.93 (6.17)	
33–36	18.01 (6.12)	
Marital status		0.139
Not married	18.26 (6.38)	
Married	17.45 (6.02)	
Divorced or separated	20.14 (7.69)	
Household		0.005
Single	18.43 (6.50)	
Cohabit	17.79 (6.08)	
Education		0.055
High school or less	18.64 (6.55)	
College	18.01 (6.24)	
Graduate level	17.94 (6.55)	
Homeownership		<0.001
Owner	17.25 (5.74)	
Non-owner	18.39 (6.34)	
Subjective income level		<0.001
1–2 (very low)	23.67 (8.42)	
3–4 (low)	19.37 (6.29)	
5–6 (intermediate)	16.97 (5.42)	
7–8 (high)	15.96 (5.04)	
9–10 (very high)	17.05 (5.50)	

Workplace bullying was also significantly associated with depressive symptoms. Workers who had experienced workplace bullying reported a higher mean CES-D-11 score (20.83) than those with no such experiences (17.72). Additionally, the number of employees in a workplace was negatively correlated with CES-D-11 scores, indicating that individuals in larger workplaces tended to report lower levels of depressive symptoms. Gender differences in CES-D-11 scores were evident, with females exhibiting significantly higher scores than males. Although age, marital status, and educational background were not significantly associated with depressive symptoms, household composition was found to be significantly associated (*p* < 0.01). Individuals living alone had higher CES-D-11 scores (18.43) than those living in multiperson households (17.79). Homeownership status also demonstrated a significant association, with homeowners reporting fewer depressive symptoms (17.25) than non-homeowners (18.39). Lastly, subjective income level was inversely associated with CES-D-11 scores, with higher income levels corresponding to fewer depressive symptoms (*p* < 0.001).

### 3.3 Multivariate regression model predicting depressive symptoms

Given that the outcome variable was count-based, Poisson regression and negative binomial regression were considered as potential modeling approaches. A key criterion for selecting an appropriate regression technique is the presence of overdispersion, in which the variance of the outcome variable exceeds its mean. Poisson regression assumes equidispersion (for example, the mean and variance are equal), whereas negative binomial regression accounts for overdispersion. To address the dispersion in our data, we compared the mean and variance of CES-D-11 scores, which were found to be 18.03 and 39.10, respectively. As the variance was substantially larger than the mean, negative binomial regression was determined to be the appropriate modeling technique for this study. Furthermore, model fit was assessed using the Pearson chi-square test. The Pearson chi-square statistic was 1,451.07 with degrees of freedom, yielding a *p*-value of 0.625. Since the null hypothesis of adequate model fit cannot be rejected at a 0.05 significance level, this indicated that the negative binomial regression model provided a good fit for the data (Table 3).

**Table 3:** Multivariate model of negative binomial regression: predicting depressive symptoms (CESD-11).

Variable	Depression	
	Estimate	IRR (95% CI)
Loneliness		
34 < (reference)		1
35–49	0.21***	1.24 (1.20, 1.28)
50–64	0.44***	1.55 (1.49, 1.61)
65=>	0.66***	1.94 (1.74, 2.16)
Commute time (min)		
30 < (reference)		1
30 =>	−0.01	0.98 (0.96, 1.01)
Work satisfaction		
1 (reference)		1
2	−0.01*	0.91 (0.83, 0.98)
3	−0.14***	0.86 (0.80, 0.93)
4	−0.19***	0.82 (0.76, 0.89)
5	−0.16***	0.84 (0.77, 0.93)
Job–major alignment		
Not at all (reference)		1
Somewhat	−0.00	0.99 (0.96, 1.02)
Perfect	0.01	1.00 (0.97, 1.05)
Experience of workplace bullying		
No (reference)		1
Yes	0.08***	1.09 (1.04, 1.14)
Number of employees		
1–4 (reference)		1
5–299	−0.01	0.98 (0.95, 1.03)
300 or more	−0.03	0.97 (0.93, 1.01)
Gender		
Female (reference)		1
Male	−0.03*	0.96 (0.94, 0.99)
Age		
18–25 (reference)		1
26–29	−0.02	0.97 (0.92, 1.02)
30–32	−0.03	0.98 (0.93, 1.03)
33–36	−0.04	0.97 (0.91, 1.02)
Marital status		
Not married (reference)		1
Married	0.04	1.04 (0.99, 1.08)
Divorced or separated	−0.01	0.98 (0.84, 1.15)
Household		
Cohabit (reference)		1
Single	0.02	1.01 (0.98, 1.05)
Education		
High school or less (reference)		1
College	−0.02	0.97 (0.93, 1.01)
Graduate level	−0.01	0.98 (0.92, 1.04)
Homeownership		
Non-owner (reference)		1
Owner	−0.03*	1.02 (0.98, 1.05)
Subjective income level		
1–2 (reference)		1
3–4	−0.08**	0.92 (0.87, 0.97)
5–6	−0.12***	0.87 (0.83, 0.92)
7–8	−0.14***	0.86 (0.81, 0.91)
9–10	0.01	1.01 (0.90, 1.14)

*p < 0.05; **p < 0.01; ***p < 0.001.

Loneliness was identified as a significant predictor of depressive symptoms, with all dummy variables for loneliness statistically significant at the 0.001 level. To facilitate interpretation, regression coefficients were transformed into incidence rate ratios (IRRs). The association between loneliness and depressive symptoms followed a linear pattern based on these IRR estimates. Compared to the reference group (scores <34, classified as the “normal” group), individuals classified as “weak intermediately lonely” (scores 35–49) had a 24% higher incidence rate of depressive symptoms (IRR: 1.24, 95% CI: 1.20, 1.28). Those in the “lonely” category (scores 50–64) exhibited a 55% higher incidence rate than the reference category (IRR: 1.55, 95% CI: 1.49, 1.61). Furthermore, individuals classified as “very lonely” (scores ≥ 65) had a 94% higher likelihood of experiencing depressive symptoms compared to the reference group (IRR: 1.94, 95% CI: 1.74, 2.16). Commuting time and number of employees were not significantly associated with depressive symptoms at a 0.05 significance level. However, job satisfaction was negatively associated with depression, with lower job satisfaction corresponding to a higher incidence of depressive symptoms. Compared to employees with the lowest job satisfaction levels, those in the second-lowest job satisfaction category exhibited a 9% lower incidence rate (IRR: 0.91, 95% CI: 0.83, 0.98). Similarly, the incidence rate was 14% lower for those with “intermediate” job satisfaction (IRR: 0.86, 95% CI: 0.80, 0.93), 18% lower for those with “high” job satisfaction (IRR: 0.82, 95% CI: 0.76, 0.89), and 16% lower for those with “very high” job satisfaction (IRR: 0.84, 95% CI: 0.77, 1.14). Job-major alignment is not a significant predictor of depressive symptoms. However, experiences of workplace bullying were significantly associated with depressive symptoms, with employees who reported workplace bullying having a 9% higher incidence rate of depressive symptoms compared to those with no such experiences (IRR: 1.09, 95% CI: 1.01, 1.14).

Significant differences related to gender were observed in depressive symptoms, indicating that male employees had a lower incidence rate than their female counterparts. An IRR of 0.96 suggests that the incidence rate of depressive symptoms among male employees was 4% lower than that among female employees. Additionally, subjective income level was strongly associated with depressive symptoms. Higher subjective income levels were associated with lower CES-D-11 scores, with the most notable difference observed in the fourth income category (7, 8). Specifically, individuals in this category had a 14% lower incidence rate of depressive symptoms compared with the reference group (lowest subjective income level).

## 4 Discussion

This study examined the association between workplace factors and psychosocial conditions and depressive symptoms among young workers.

The results confirm that loneliness is a significant predictor of depressive symptoms, with a clear dose–response relationship observed between loneliness levels and CES-D-11 scores. Individuals experiencing higher levels of loneliness reported significantly greater depressive symptom severity, consistent with previous research on the detrimental effects of social isolation on mental health (45, 46). These findings reinforce the importance of social connectivity as a critical component of psychological well-being (47, 48).

Job satisfaction demonstrated a strong inverse relationship with depressive symptoms. Employees who reported higher levels of job satisfaction exhibited significantly lower CES-D-11 scores, consistent with existing literature highlighting the protective role of job engagement and positive work experiences in mental health (38, 49). These findings provide broader evidence that satisfaction with the workplace environment is closely related to reduced psychological distress.

While job-major alignment initially appeared to be associated with depressive symptoms in the unadjusted models, multivariate analysis did not confirm its significance as an independent predictor. This suggests that the relationship between educational background and job fit may be more complex and mediated by other factors influencing employee well-being.

Workplace bullying emerged as a consistent and significant predictor of depressive symptoms. Employees reporting experiences of bullying exhibited greater depressive symptom severity, in line with the CATS, which posits that chronic exposure to negative workplace interactions may lead to emotional exhaustion and elevated risk of psychological distress (26–30). This emphasizes the need to address interpersonal dynamics in occupational mental health research.

Contrary to previous findings, commute time was not a significant predictor of depressive symptoms in this study. While long commuting times have previously been associated with stress and fatigue, other factors such as transportation quality, flexibility in work arrangements, and personal coping strategies may mediate this relationship (50).

Socioeconomic factors, including subjective income level and homeownership status, are also associated with depressive symptoms. Higher income levels and homeownership were associated with lower CES-D-11 scores, possibly reflecting the stabilizing influence of financial security on psychological health.

Finally, notable gender differences were observed, with male employees reporting lower depressive symptom scores than female employees.

## 5 Implications

These findings have several meaningful implications for workplace mental health strategies. Job satisfaction has emerged as a strong factor associated with reduced depressive symptoms (51–54). As job satisfaction arises from workplace conditions, it may function as a psychological asset that supports mental health. Conversely, job dissatisfaction may be associated with an increased psychological burden and distress.

To foster job satisfaction, organizations should implement participatory management strategies that encourage employee engagement in decision-making processes (55–58). Transforming communication patterns from one-way, top-down directives to interactive, two-way exchanges can enhance employees’ sense of control and belonging, which may in turn be associated with improved mental health outcomes.

Another strategy is the proactive adoption of employee assistance programs (EAPs) to support employees’ mental well-being. EAPs, offering psychological counseling and emotional support services, may be associated with reduced stress and better coping mechanisms (59–61). Stress remains a major challenge in workplace mental health, and minimizing stress is critical for fostering healthier individuals and organizations.

Workplace interventions should adapt to context-specific challenges. In South Korea, the competitive educational and work environments contribute to elevated stress levels. Organizations may benefit from introducing flexible work arrangements, stress management workshops, and resilience-building programs to alleviate workplace strain (61–65). Intervention programs at the individual level, such as psychological counseling, physical exercise, and meditation, as well as organizational-level strategies, such as job redesign and flexible work hours, have shown promise in reducing workplace stress (62, 63, 66–68).

Additionally, loneliness has been identified as an important contributor to depressive symptoms in the workplace. Social disconnection, particularly among remote workers, can exacerbate mental health risks. In line with Wong et al. (69), efforts to increase employee voices, restructure work to enhance social connections, and foster supportive peer networks could be effective strategies to mitigate loneliness and promote better mental health outcomes.

Therefore, future workplace mental health initiatives should not only address traditional job demands but also prioritize enhancing social connectedness, improving job satisfaction, and reducing interpersonal stressors, such as bullying, to create more supportive and psychologically healthy work environments.

## 6 Conclusion

Our findings suggest that job satisfaction is negatively related to depressive symptoms, whereas workplace bullying is associated with higher levels of depressive symptoms. In contrast, job-major alignment and commute time did not show strong associations with depressive symptoms, indicating that factors such as autonomy, compensation, and workplace social dynamics may be more closely linked to mental well-being among young employees. These findings imply that workplace psychosocial interventions may target psychological safety, stress management, and employee engagement to support their mental well-being.

## 7 Limitation and research directions

While this study emphasizes the importance of workplace conditions for employees’ mental health, several methodological issues should be addressed. First, we relied on public health survey data, which may be subject to nonresponse errors unless the nonresponse rate is zero (for example, a 100% response rate). According to Groves (70), a nonresponse error consists of two components: the response rate and the difference in reporting values between respondents and nonrespondents. In most cases, the second component could not be estimated, because no information was available from those who did not participate in the survey. Alternatively, nonresponse can be approximated using the response rate of the first component. The higher the response rate, the lower is the potential for nonresponse error. Given that our dataset had a response rate of approximately 57%, it is likely that a nonresponse error affected our findings. Future studies should address this issue by maximizing the survey response rates. Second, the reliance on self-reported data may introduce reporting bias. Recall bias may occur when respondents attempt to remember past behaviors (71), and social desirability bias can lead individuals to overreport socially acceptable behaviors and underreport socially undesirable behaviors. Such bias is a critical concern in survey research (72). Moreover, the use of self-reported data in regression analysis can result in common method bias (73). Although we assessed this issue using Harman’s single-factor analysis and found that the variance explained by the first factor was below the threshold, this does not guarantee that our results are free from common method bias. Third, we employed a cross-sectional survey design, which limited causal inference because all variables were measured simultaneously. To identify causal relationships, the temporal order of variables must be established. Therefore, the relationships observed in this study should be interpreted as associations, rather than causal effects. Future research should employ longitudinal designs that incorporate time lags between work-related factors and outcomes to clarify the temporal sequence of workplace factors and depressive symptoms. Fourth, we measured the multidimensional concept of job satisfaction using a single survey item and assessed workplace-specific loneliness using general loneliness measurement items. Future research should employ multiple items to capture various dimensions of job satisfaction and develop measures specifically designed to assess workplace-related loneliness. Finally, this study focused solely on young workers in Seoul, which limits the generalizability of the findings to the broader Korean workforce. Workplace experiences may differ across regions in South Korea owing to cultural, economic, and industry-specific variations. Therefore, the results should be interpreted with caution.

Considering these limitations, researchers should aim to minimize total survey error (74, 75). Longitudinal research designs and analyses of sector-specific differences in workplace mental health outcomes could enhance the robustness and generalizability of research findings. Nationwide studies encompassing young workers from all the regions of South Korea would provide a more comprehensive picture. Expanding this research to include young workers in other countries could also yield valuable cross-cultural insights into how workplace conditions affect mental well-being in different social and economic contexts.

## Data Availability

Publicly available datasets were analyzed in this study. This data can be found here: https://syps.si.re.kr/homepage/reference/data/view/18.

## References

[1] Daoud H Sellami I Ben Chabene C Ghrab MA Haddar A Hajjaji M. The impact of working conditions on the mental health of workers in a confectionery factory. Eur Psychiatry. (2024) 67:S596. doi: 10.1192/j.eurpsy.2024.1241, PMID: 38385431

[2] Johnson A Dey S Nguyen H Groth M Joyce S Tan L. A review and agenda for examining how technology-driven changes at work will impact workplace mental health and employee well-being. Aust J Manag. (2020) 45:402–24. doi: 10.1177/0312896220922292

[3] Henke RM. Knowing well, being well: well-being born of understanding: supporting workforce mental health during the pandemic. Am J Health Promot. (2022) 36:1213–44. doi: 10.1177/08901171221112488, PMID: 36003017 PMC9523433

[4] Moe-Byrne T Shepherd J Merecz-Kot D Sinokki M Naumanen P Hakkaart-van Roijen L. Effectiveness of tailored digital health interventions for mental health at the workplace: a systematic review of randomised controlled trials. PLoS Digit Health. (2022) 1:e0000123. doi: 10.1371/journal.pdig.0000123, PMID: 36812547 PMC9931277

[5] de Oliveira C Saka M Bone L Jacobs R. The role of mental health on workplace productivity: a critical review of the literature. Appl Health Econ Health Policy. (2023) 21:167–93. doi: 10.1007/s40258-022-00761-w, PMID: 36376610 PMC9663290

[6] Kelloway EK Dimoff JK Gilbert S. Mental health in the workplace. Annu Rev Organ Psychol Organ Behav. (2023) 10:363–87. doi: 10.1146/annurev-orgpsych-120920-050527

[7] Ganson KT Tsai AC Weiser SD Benabou SE Nagata JM. Job insecurity and symptoms of anxiety and depression among U.S. young adults during COVID-19. J Adolesc Health. (2021) 68:53–6. doi: 10.1016/j.jadohealth.2020.10.008, PMID: 33183926

[8] Law PCF Too LS Butterworth P Witt K Reavley N Milner AJ. A systematic review on the effect of work-related stressors on mental health of young workers. Int Arch Occup Environ Health. (2020) 93:611–22. doi: 10.1007/s00420-020-01516-7, PMID: 31932956

[9] Milner A Krnjacki L LaMontagne AD. Psychosocial job quality and mental health among young workers: a fixed-effects regression analysis using 13 waves of annual data. Scand J Work Environ Health. (2017) 43:50–8. doi: 10.5271/sjweh.3608, PMID: 27918610

[10] Afonso P Fonseca M Pires JF. Impact of working hours on sleep and mental health. Occup Med (Lond). (2017) 67:377–82. doi: 10.1093/occmed/kqx054, PMID: 28575463

[11] Bannai A Tamakoshi A. The association between long working hours and health: a systematic review of epidemiological evidence. Scand J Work Environ Health. (2014) 40:5–18. doi: 10.5271/sjweh.3388, PMID: 24100465

[12] Bentley RJ Kavanagh A Krnjacki L LaMontagne AD. A longitudinal analysis of changes in job control and mental health. Am J Epidemiol. (2015) 182:328–34. doi: 10.1093/aje/kwv046 26138706

[13] Wang ML Narcisse MR Togher K McElfish PA. Job flexibility, job security, and mental health among US working adults. JAMA Netw Open. (2024) 7:e243439. doi: 10.1001/jamanetworkopen.2024.3439, PMID: 38526492 PMC10964112

[14] Kim SY Shin YC Oh KS Shin DW Lim WJ Cho SJ. Association between work stress and risk of suicidal ideation: a cohort study among Korean employees examining gender and age differences. Scand J Work Environ Health. (2020) 46:198–208. doi: 10.5271/sjweh.3852, PMID: 31539082

[15] Lee H Cho SJ Shin YC Shin DW Park JH Kim M. Suicidal ideation and long work hours by gender in Korean employees: the Kangbuk Samsung workplace mental health study: a cross-sectional study. Precis Fut Med. (2022) 6:177–87. doi: 10.23838/pfm.2022.00086

[16] Lee KH Ho Chae C Ouk Kim Y Seok Son J Kim JH Woo Kim C. Anxiety symptoms and occupational stress among young Korean female manufacturing workers. Ann Occup Environ Med. (2015) 27:24. doi: 10.1186/s40557-015-0075-y, PMID: 26568830 PMC4644331

[17] Daily K. Experiencing workplace bullying (2015). Available online at: https://www.khan.co.kr/article/201512202211515 (Accessed January 15, 2025).

[18] News. Workplace bullying case of the last year…the highest. 1 (2025). Available online at: https://www.news1.kr/economy/employment-labor/5679796 (Accessed January 15, 2025)

[19] TBS. Antidepressant prescriptions soar 65% in 10 years… Nearly quadrupled in 20-somethings (2024). Available online at: https://tbs.seoul.kr/news/newsView.do?seq_800=20521965 (Accessed February 10, 2025).

[20] Chosun Daily. “Hospital bedside attendance with depression doubles in 3 years”. A10 (2025). Available online at: https://www.chosun.com/national/education/2025/02/12/G726IOL5X5BM5KUN5CCSEMRUEM/ (Accessed February 20, 2025).

[21] Bakker AB Demerouti E. The job demands-resources model: state of the art. J Manag Psychol. (2007) 22:309–28. doi: 10.1108/02683940710733115

[22] Demerouti E Bakker AB Nachreiner F Schaufeli WB. The job demands-resources model of burnout. J Appl Psychol. (2001) 86:499–512. doi: 10.1037/0021-9010.86.3.499 11419809

[23] Duan-Porter W Hatch D Pendergast JF Freude G Rose U Burr H. 12-month trajectories of depressive symptoms among nurses-contribution of personality, job characteristics, coping, and burnout. J Affect Disord. (2018) 234:67–73. doi: 10.1016/j.jad.2018.02.090, PMID: 29522946 PMC6087547

[24] Oliveira AM Silva MT Galvão TF Lopes LC. The relationship between job satisfaction, burnout syndrome and depressive symptoms: an analysis of professionals in a teaching hospital in Brazil. Medicine. (2018) 97:e13364. doi: 10.1097/MD.0000000000013364, PMID: 30544404 PMC6310545

[25] Bakker AB Demerouti E Sanz-Vergel A. Job demands–resources theory: ten years later. Annu Rev Organ Psychol Organ Behav. (2023) 10:25–53. doi: 10.1146/annurev-orgpsych-120920-053933

[26] Meurs JA Perrewé PL. Cognitive activation theory of stress: an integrative theoretical approach to work stress. J Manag. (2011) 37:1043–68. doi: 10.1177/0149206310387303, PMID: 40372777

[27] Ursin H Eriksen HR. The cognitive activation theory of stress. Psychoneuroendocrinology. (2004) 29:567–92. doi: 10.1016/S0306-4530(03)00091-X, PMID: 15041082

[28] Du Preez A Law T Onorato D Lim YM Eiben P Musaelyan K. The type of stress matters: repeated injection and permanent social isolation stress in male mice have a differential effect on anxiety- and depressive-like behaviours, and associated biological alterations. Transl Psychiatry. (2020) 10:325. doi: 10.1038/s41398-020-01000-3, PMID: 32958745 PMC7505042

[29] Stewart C Rogers F Pilch M Stewart I Barnes-Holmes Y Westermann S. The effect of social exclusion on state paranoia and explicit and implicit self-esteem in a non-clinical sample. J Behav Ther Exp Psychiatry. (2017) 57:62–9. doi: 10.1016/j.jbtep.2017.04.001, PMID: 28419917

[30] Yun JY Shim G Jeong B. Verbal abuse related to self-esteem damage and unjust blame harms mental health and social interaction in college population. Sci Rep. (2019) 9:5655. doi: 10.1038/s41598-019-42199-6, PMID: 30948757 PMC6449380

[31] Lieberz J Shamay-Tsoory SG Saporta N Kanterman A Gorni J Esser T. Behavioral and neural dissociation of social anxiety and loneliness. J Neurosci. (2022) 42:2570–83. doi: 10.1523/JNEUROSCI.2029-21.2022, PMID: 35165170 PMC8944238

[32] Weber M Schulze L Bolzenkötter T Niemeyer H Renneberg B. Mental health and loneliness in university students during the COVID-19 pandemic in Germany: a longitudinal study. Front Psych. (2022) 13:848645. doi: 10.3389/fpsyt.2022.848645, PMID: 35492687 PMC9051079

[33] Devonish D. Dangers of workplace bullying: evidence from the Caribbean. J Aggression Conflict Peace Res. (2017) 9:69–80. doi: 10.1108/JACPR-05-2016-0228

[34] Sprigg CA Niven K Dawson J Farley S Armitage CJ. Witnessing workplace bullying and employee well-being: a two-wave field study. J Occup Health Psychol. (2019) 24:286–96. doi: 10.1037/ocp0000137, PMID: 30489100

[35] Helm PJ Medrano MR Allen JJB Greenberg J. Existential isolation, loneliness, depression, and suicide ideation in young adults. J Soc Clin Psychol. (2020) 39:641–74. doi: 10.1521/jscp.2020.39.8.641

[36] McClelland H Evans JJ Nowland R Ferguson E O’Connor RC. Loneliness as a predictor of suicidal ideation and behaviour: a systematic review and meta-analysis of prospective studies. J Affect Disord. (2020) 274:880–96. doi: 10.1016/j.jad.2020.05.004, PMID: 32664029

[37] Lopes H Santos T Silva C Lima A Rocha L. Relationship between depressive symptoms, burnout, job satisfaction and patient safety culture among workers at a university hospital in the Brazilian context. Sao Paulo Med J. (2022) 140:631–8. doi: 10.1590/1516-3180.2021.0712.R1.29062022 PMC9671242 35508009

[38] Yang S Kim JH Jung M Kim HC Leem JH Park SG. Effect of job satisfaction on depression after adjusting for satisfaction with other life domains. Ann Occup Environ Med. (2024) 36:e8. doi: 10.35371/aoem.2024.36.e8, PMID: 38623262 PMC11016776

[39] Neto M Ferreira AI Martinez LF Ferreira PC. Workplace bullying and presenteeism: the path through emotional exhaustion and psychological wellbeing. Ann Work Exposures Health. (2017) 61:528–38. doi: 10.1093/annweh/wxx022, PMID: 28371824

[40] Lo Presti AL Pappone P Landolfi A. The associations between workplace bullying and physical or psychological negative symptoms: anxiety and depression as mediators. Eur. J Psychol. (2019) 15:808–22. doi: 10.5964/ejop.v15i4.1733, PMID: 33680161 PMC7909204

[41] Radloff LS. The CES-D scale: a self-report depression scale for research in the general population. Appl Psychol Meas. (1977) 1:385–401. doi: 10.1177/014662167700100306

[42] Jiang L Wang Y Zhang Y Li R Wu H Li C. The reliability and validity of the center for epidemiologic studies depression scale (CES-D) for Chinese university students. Front Psych. (2019) 10:315. doi: 10.3389/fpsyt.2019.00315, PMID: 31178764 PMC6537885

[43] Park EY Kim JH. Unidimensionality and measurement invariance of the 11-item Korean CES-D scale among examining parents of individuals with cerebral palsy. J Child Fam Stud. (2020) 29:895–903. doi: 10.1007/s10826-019-01630-2

[44] Russell D. UCLA loneliness scale (version 3): reliability, validity, and factor structure. J Pers Assess. (2010) 66:20–40. doi: 10.1207/s15327752jpa6601_2, PMID: 8576833

[45] Lee SL Pearce E Ajnakina O Johnson S Lewis G Mann F. The association between loneliness and depressive symptoms among adults aged 50 years and older: a 12-year population-based cohort study. Lancet Psychiatry. (2021) 8:48–57. doi: 10.1016/S2215-0366(20)30383-7, PMID: 33181096 PMC8009277

[46] Park NS Lee BS Chiriboga DA Chung S. Loneliness as a mediator in the relationship between social engagement and depressive symptoms: age differences among community-dwelling Korean adults. Health Soc Care Community. (2019) 27:706–16. doi: 10.1111/hsc.12687, PMID: 30485596

[47] Green MJ Whitley E Niedzwiedz CL Shaw RJ Katikireddi SV. Social contact and inequalities in depressive symptoms and loneliness among older adults: a mediation analysis of the English longitudinal study of ageing. SSM Popul Health. (2021) 13:100726. doi: 10.1016/j.ssmph.2021.100726, PMID: 33521227 PMC7820553

[48] Son H Cho HJ Cho S Ryu J Kim S. The moderating effect of social support between loneliness and depression: differences between the young-old and the old-old. Int J Environ Res Public Health. (2022) 19:2322. doi: 10.3390/ijerph19042322, PMID: 35206508 PMC8871923

[49] Nakata A. Long working hours, job satisfaction, and depressive symptoms: a community-based cross-sectional study among Japanese employees in small- and medium-scale businesses. Oncotarget. (2017) 8:53041–52. doi: 10.18632/oncotarget.18084, PMID: 28881792 PMC5581091

[50] Knott CS Panter J Foley L Ogilvie D. Changes in the mode of travel to work and the severity of depressive symptoms: a longitudinal analysis of UK biobank. Prev Med. (2018) 112:61–9. doi: 10.1016/j.ypmed.2018.03.018, PMID: 29604327 PMC5999356

[51] Emmanuel Olatunde B Odusanya O. Job satisfaction and psychological well-being among mental health nurses. IJTCM. (2015) 3:64–70. doi: 10.19070/2333-8385-1500012, PMID: 40336382

[52] Gechman AS Wiener Y. Job involvement and satisfaction as related to mental health and personal time devoted to work. J Appl Psychol. (1975) 60:521–3. doi: 10.1037/h0076902, PMID: 1176425

[53] Nadinloyi KB Sadeghi H Hajloo N. Relationship between job satisfaction and employees mental health. Procedia Soc Behav Sci. (2013) 84:293–7. doi: 10.1016/j.sbspro.2013.06.554

[54] Wright TA Bonett DG. Job satisfaction and psychological well-being as nonadditive predictors of workplace turnover. J Manag. (2007) 33:141–60. doi: 10.1177/0149206306297582, PMID: 40372777

[55] Akhter S Saha SP Mahfuz I. Participatory management and job satisfaction: a case of Bangladesh. World J Manag. (2019) 10:45–56.

[56] Hyman J Mason B. Managing employee involvement and participation. London: Sage Publications (1995).

[57] Kim S. Participative management and job satisfaction: lessons for management leadership. Public Admin Rev. (2002) 62:231–41. doi: 10.1111/0033-3352.00173

[58] Zhu Y Xie Y Warner M Guo Y. Employee participation and the influence on job satisfaction of the ‘new generation’ of Chinese employees. Int J Hum Resour Manag. (2015) 26:2395–411. doi: 10.1080/09585192.2014.990397

[59] Bradley JR Sutherland V. Stress management in the workplace: taking employees’ views into account. Empl Couns Today. (1994) 6:4–9. doi: 10.1108/13665629410060443

[60] Tran CTH Tran HTM Nguyen HTN Mach DN Phan HSP Mujtaba BG. Stress management in the modern workplace and the role of human resource professionals. Bus Ethics Leadersh. (2020) 4:26–40. doi: 10.21272/bel.4(2).26-40.2020

[61] Panigrahi A. Managing stress at workplace. J Manag Res Anal. (2016) 3:154–60. doi: 10.18231/2394-2770.2016.0001

[62] Holman D Johnson S O’Connor E. Stress management interventions: improving subjective psychological well-being in the workplace In: Diener E Oishi S Tay L, editors. Handbook of well-being. nobascholar.com. Salt Lake City: DEF Publishers (2018)

[63] Jacobs S Johnson S Hassell K. Managing workplace stress in community pharmacy organisations: lessons from a review of the wider stress management and prevention literature. Int J Pharm Pract. (2018) 26:28–38. doi: 10.1111/ijpp.12360, PMID: 28349623

[64] Mimura C Griffiths P. The effectiveness of current approaches to workplace stress management in the nursing profession: an evidence based literature review. Occup Environ Med. (2003) 60:10–5. doi: 10.1136/oem.60.1.10, PMID: 12499451 PMC1740380

[65] Tetrick LE Winslow CJ. Workplace stress management interventions and health promotion. Annu Rev Organ Psychol Organ Behav. (2015) 2:583–603. doi: 10.1146/annurev-orgpsych-032414-111341

[66] Semmer NK. Job stress interventions and the organization of work. Scand J Work Environ Health. (2006) 32:515–27. doi: 10.5271/sjweh.1056, PMID: 17173207

[67] Bond FW Bunce D. Job control mediates change in a work reorganization intervention for stress reduction. J Occup Health Psychol. (2001) 6:290–302. doi: 10.1037//1076-8998.6.4.290, PMID: 11605824

[68] Humphrey SE Nahrgang JD Morgeson FP. Integrating motivational, social, and contextual work design features: a meta-analytic summary and theoretical extension of the work design literature. J Appl Psychol. (2007) 92:1332–56. doi: 10.1037/0021-9010.92.5.1332, PMID: 17845089

[69] Wong YJ Qureshi A Mahendran R Verma SK. Workplace loneliness and employee mental health: a multi-level meta-analysis. BMJ Open. (2023) 12:e066389. doi: 10.1136/bmjopen-2022-066389, PMID: 36600336 PMC9743407

[70] Groves RM. Nonresponse rates and nonresponse bias in household surveys. Pub Opin Q. (2006) 70:646–75. doi: 10.1093/poq/nfl033

[71] Tourangeau R Rips LJ Rasinki K. The psychology of survey response. London: Cambridge University Press (2000).

[72] Fisher RJ. Social desirability bias and the validity of indirect questioning. J Cons Res. (1993) 20:303–15. doi: 10.1086/209351

[73] Podsakoff PM Mackenzie SB Lee J-Y Podsakoff NP. Common method biases in behavioral research: a critical review of the literature and recommended remedies. J Appl Psychol. (2003) 88:879–903. doi: 10.1037/0021-9010.88.5.879, PMID: 14516251

[74] Groves RM Fowler FJ Jr Couper MP Lepkowski JM Singer E Tourangeau R. Survey methodology. New York: John Wiley & Sons (2009).

[75] Lee G Benoit-Bryan J Johnson TP. Survey research in public administration: assessing mainstream journals with a total survey error framework. Pub Adm Rev. (2011) 41:168–84. doi: 10.1111/j.1540-6210.2011.02482.x

